# A Cascaded Approach for Correcting Ionospheric Contamination with Large Amplitude in HF Skywave Radars

**DOI:** 10.1155/2014/693872

**Published:** 2014-01-20

**Authors:** Yajun Li, Yinsheng Wei, Rujiang Guo, Rongqing Xu, Zhuoqun Wang, Xiudong Tang

**Affiliations:** School of Electronics and Information Engineering, Harbin Institute of Technology, Harbin 150001, China

## Abstract

Ionospheric phase perturbation with large amplitude causes broadening sea clutter's Bragg peaks to overlap each other; the performance of traditional decontamination methods about filtering Bragg peak is poor, which greatly limits the detection performance of HF skywave radars. In view of the ionospheric phase perturbation with large amplitude, this paper proposes a cascaded approach based on improved S-method to correct the ionospheric phase contamination. This approach consists of two correction steps. At the first step, a time-frequency distribution method based on improved S-method is adopted and an optimal detection method is designed to obtain a coarse ionospheric modulation estimation from the time-frequency distribution. At the second correction step, based on the phase gradient algorithm (PGA) is exploited to eliminate the residual contamination. Finally, use the measured data to verify the effectiveness of the method. Simulation results show the time-frequency resolution of this method is high and is not affected by the interference of the cross term; ionospheric phase perturbation with large amplitude can be corrected in low signal-to-noise (SNR); such a cascade correction method has a good effect.

## 1. Introduction

HF skywave radars use the ionospheric reflection to realize space or sea target detection. Since the ionosphere is a time-varying, nonstationary, dispersion, layered lossy medium, the hierarchical structure and the nonstationary characteristics make shortwave signal phase path produce linear and nonlinear changes and multimode propagation effects, thereby causing sea clutter spectrum frequency shift and broadening. The broadening of clutter spectrum is very easy to overwhelm slow moving ship target, thus affecting the radar detection performance. Multimode and multipath propagation effects can usually be solved by the frequency monitoring system (namely, real-time single-mode transmission), while the perturbation of signal phase path generated by ionosphere is generally unpredictable, and the levels of contamination are not the same.

Ionospheric phase contamination causes sea clutter spectrum broadening; if the phase contamination function could be extracted from the broaden clutter, we can construct the required correction function. The echo spectrum can be sharpened by using the obtained correction function to improve the radar target detection performance. At present, many methods have been proposed for extracting ionospheric phase contamination from broadening sea clutter, such as the maximum entropy spectral estimation method, the pseudo-Wigner-Ville distribution (PWVD), piecewise polynomial phase modeling, PGA, and minimum entropy search method [1–5]. These methods are firstly required to extract the broadening Bragg peak to estimate instantaneous frequency. When amplitude of ionospheric phase perturbation is small, the broadening extent of two first-order Bragg peaks is small; we can use a band-pass filter to filter out a Bragg peak. However, in the case of large phase contamination, the two broadening Bragg peaks will overlap; we cannot use the band-pass filter to filter out a Bragg peak for analysis, making the effect of ionospheric phase decontamination too poor to be corrected.

In view of this ionospheric phase perturbation with large amplitude, the current preferred method is to study the time-frequency distribution and sharpen echo spectrum. The most commonly used time-frequency distribution techniques are short-time Fourier transform (STFT), Wigner-Ville distribution (WVD), PWVD, and other methods. The advantage of STFT has no cross term between signals, meeting the property of linear additive; however its ability of time-frequency concentration is poor. WVD has perfect time-frequency concentration, but it has serious cross-time interference. PWVD method is simple but works well for high SNR. Compared to STFT and WVD, Stankovic proposed S-method which has the advantage of both [6], as long as the value of *L* is reasonably selected; it can achieve either the non-cross term, or the good time-frequency concentration. However, as the length of the window function becomes longer, S-method distribution will become a more unavoidable cross term, with frequency concentration of auto terms becoming better. As the length of the window function becomes shorter, S-method distribution will avoid producing cross terms, but not as good as the former frequency concentration [6, 7].

Considering the difficulties of the window function selection as well as the good time-frequency concentration and free of cross term, we proposed a new S^2^-method time-frequency method to detect the HF radar maneuvering targets in literature [8]. In this paper, we further study and improve this new S^2^-method and apply it to correct ionospheric contamination for HF skywave radar. Firstly, the basic principle of the cascaded approach based on S^2^-method for correcting ionospheric contamination is given. Then the instantaneous frequency is extracted by optimal path detection, and the performance of decontamination of this method in the case of ionospheric phase perturbation with large amplitude and low SNR is simulated and analyzed. Finally, the effectiveness of the method is verified with measured data. Simulation results show that the time-frequency resolution of this method is high, without cross term interference, and this method can correct ionospheric phase perturbation with large amplitude under low SNR.

## 2. A Cascaded Approach Based on Improved S-Method for Correcting Ionospheric Contamination

### 2.1. The Basic Principle of S-Method Algorithm

The time-frequency methods based on WVD class are susceptibe to interference of cross term. Although there is no cross term in STFT, its time-frequency concentration is poor. S-method was proposed by Stankovic [6], which combines the advantages of spectrogram (SPEC) and WVD, while maintaining the time-frequency resolution of WVD and reducing its cross term, and holds strong antinoise performance and low computational complexity.

S-method distribution of discrete signal *x*(*n*) was defined as [6–8]

(1) $$\begin{array}{c} \text{SM}\left(n,k\right)=\frac{1}{N+1}\overset{L}{\underset{i=-L}{\sum }}\text{STFT}\left(n,k+i\right)\text{STF}{\text{T}}^{*}\left(n,k-i\right); \end{array}$$

here, *k* is the frequency number, *n* is the time series, STFT(*n*, *k*) is the short-time Fourier transform of the signal *x*(*n*), *N* + 1 is the length of the signal *x*(*n*), and *L* is the computational numbers of items.

S-method distribution of discrete signal *x*(*n*) can also be calculated by the following formula:

(2) SM(n,k)=|STFT(t,ω)|2 +2Re{STFT(n,k+1)·STFT∗(n,k−1)} +2Re{STFT(n,k+2)·STFT∗(n,k−2)} +⋯+2Re{STFT(n,k+L)·STFT∗(n,k−L)}.

If S-method distribution with L-items is:

(3) $$\begin{array}{c} \text{S}{\text{M}}_{L}\left(n,k\right)=\overset{L}{\underset{i=-L}{\sum }}\text{STFT}\left(n,k+i\right)\text{STF}{\text{T}}^{*}\left(n,k-i\right). \end{array}$$

Equation (2) shows S-method distribution can be calculated by iteration:

(4) SML(n,k)=SML−1(n,k) +2Re{STFT(n,k+L)STFT∗(n,k−L)},

wherein formula (4) is a fast calculation method of S-method distribution.

Although S-method has more advantages compared to STFT and WVD, however, in dealing with multitarget signal, the values of selected *L* are constrained by resolution and signal energy, especially when the pitch of multiple targets in frequency domain is less than the bandwidth of target. S^2^-method is the improved algorithm based on the S-method time-frequency analysis which is obtained by suppressing the cross terms between the contaminated sea clutter.

### 2.2. The Proposed S^2^-Method Algorithm

In time-frequency analysis of nonstationary multitarget signal, if the window function becomes longer, S-method time-frequency analysis will produce inevitable cross terms, but frequency concentration of autoitems will be better. In contrary when it becomes shorter, S-method time-frequency distribution can avoid producing cross terms, but frequency concentration is not as good as the former. S^2^-method algorithm can simultaneously combine the advantages of both and at the same time has good frequency concentration and does not contain cross terms.

Assuming the signal *x*(*n*) = *x*_1_(*n*) + *x*_2_(*n*) is the superposition of two Bragg peaks *x*_1_(*n*) and *x*_2_(*n*) of the sea clutter, we define the window function *w*_1_(*n*) in the following:

(5) $$\begin{array}{c} w_{1}\left(n\right)=\{\begin{array}{cc} 1, & \frac{-N}{2}\leq n\leq \frac{N}{2}\\ 0. & \end{array} \end{array}$$

The S-method distribution SM_*w*_1__(*n*, *k*) can be expressed as the combination of three parts: S-method distribution of *x*_1_(*n*), S-method distribution of *x*_2_(*n*), *x*_1_(*n*), and *x*_2_(*n*) generated cross term. And these three parts are

(6) $$\begin{array}{c} \text{S}{\text{M}}_{w_{1}}\left(n,k\right)=\text{S}{\text{M}}_{w_{1}}^{1}\left(n,k\right)+\text{S}{\text{M}}_{w_{1}}^{2}\left(n,k\right)+\text{S}{\text{M}}_{w_{1}}^{1,2}\left(n,k\right). \end{array}$$

Then select a suitable window function *w*_2_(*n*), which meets the condition that there are no cross terms by S-method for *x*(*n*); here we make *w*_2_ as hamming window, and the length of window is *N*/4; then On this condition, the S-method distribution SM_*w*_2__(*n*, *k*) of *x*(*n*) can be expressed as the sum of two parts. Consider

(7) $$\begin{array}{c} \text{S}{\text{M}}_{w_{2}}\left(n,k\right)=\text{S}{\text{M}}_{w_{2}}^{1}\left(n,k\right)+\text{S}{\text{M}}_{w_{2}}^{2}\left(n,k\right). \end{array}$$

We use SM_*w*_2__(*n*, *k*) as a mask to effectively select autoitems of SM_*w*_1__(*n*, *k*), SM_*w*_1__^1^(*n*, *k*), and SM_*w*_1__^2^(*n*, *k*), and to suppress cross term SM_*w*_1__^1,2^(*n*, *k*).

We define S^2^-method as follows:

(8) $$\begin{array}{c} {\text{SM}}^{2}\left(n,k\right)={\text{SM}}_{w_{1}}\left(n,k\right)\times \text{l}\left(\text{S}{\text{M}}_{w_{2}}\left(n,k\right)\geq \eta \right), \end{array}$$

where *l*(·) is an indicative function, *η* is a parameter determined by the noise floor, and l(SM_*w*_2__(*n*, *k*) ≥ *η*) can be expressed as

(9) $$\begin{array}{c} \text{l}\left(\text{S}{\text{M}}_{w_{2}}\left(n,k\right)\geq \eta \right)=\{\begin{array}{cc} 1, & \text{S}{\text{M}}_{w_{2}}\left(n,k\right)\geq \eta \\ 0, & \text{S}{\text{M}}_{w_{2}}\left(n,k\right)<\eta . \end{array} \end{array}$$

In this way, we can suppress the cross term of S-method; according to formulas (5) to (9), we can obtain

(10) SM2(n,k)=SMw11(n,k)+SMw12(n,k)=WD1(n,k)+WD2(n,k).

Note that the value of *η* in formula (8) is necessary to profile observe SM_*w*_2__(*n*, *k*) to determine their specific size.

### 2.3. Extract the Instantaneous Frequency by Optimal Path Detection Algorithm

The two broadening Bragg peaks can be sharpened by S^2^-method; the next step key is to extract the instantaneous frequency from the S^2^-method time-frequency distribution. There are two main methods to estimate instantaneous frequency from time-frequency distribution: the peak detection method and the first moment method. Among them, the first moment method in practice does not get good results, because the method is very strict on the SNR, and the cross term will greatly affect the performance of the first moment.

Based on the above discussion, we consider from the time-frequency distribution of sea clutter contaminated by ionospheric phase perturbation to directly extract ionospheric frequency modulation. Optimal path detection algorithm is used here to extract the instantaneous frequency from the S^2^-method distribution. Based on the time-frequency analysis for signal, literature [9] proposed an optimal path detection algorithm. The frequency of signal along the curve of time change can be portrayed by time-frequency analysis method in time-frequency domain. The so-called “best path” is the distribution curve of frequency of signal in time-frequency domain. The idea of signal detection is to adaptively search the time-frequency distribution curve of signal, then along this curve to accumulate the energy of signal; the signal energy is concentrated together, increasing the detection SNR. We need eigen decomposition for S^2^-method distribution of signal before optimal path detection.

Firstly, we need to obtain the S^2^-method distribution of contaminated signal *x*(*n*) according to the definition of formula (8), and then construct the matrix *R*:

(11) R(n1,n2) =1N+1∑k=−N/2N/2SM2(n1+n22,k)ej(2π/(N+1))k(n1−n2).

Then do the eigen decomposition for matrix *R* to obtain a set of eigenvalues and eigenvectors satisfying

(12) $$\begin{array}{c} R=\overset{M}{\underset{i=1}{\sum }}\lambda_{i}u_{i}u_{i}^{*}, \end{array}$$

wherein *u*_*i*_ is the corresponding eigenvectors and *λ*_*i*_ is the corresponding eigenvalues. We select the first *p* largest eigenvalues *λ*_*i*_, 1 < *i* < *p*, and use corresponding eigenvectors *u*_*i*_, 1 < *i* < *p*, according to formula (13) to reconstruct the target signal:

(13) $$\begin{array}{c} x_{i}\left(n\right)=\sqrt{\lambda_{wi}}u_{i}\left(n\right). \end{array}$$

If the signal *x*(*n*) contains more than one frequency component, each of the reconstructed signals *x*_*i*_(*n*) corresponds to a signal component, so that the multitarget signal can be separated.

The optimal path detection algorithm to extract the instantaneous frequency is given in the following steps.

 Step one: the sea clutter is analyzed by S^2^-method time-frequency distribution SM^2^[·].
 Step two: SM^2^[·] is done by eigen decomposing, and the signal *x*_*i*_(*n*), *i* = 1,2,…, is reconstructed.
 Step three: the *x*_*i*_(*n*), *i* = 1,2,…, is, respectively, made S^2^-method distribution SM^2^[·], using the optimal path detection method for each SM_*i*_^2^[·] for detection; the judgment result is obtained.
 Step four: using the extracted instantaneous frequency, the phase correction function is obtained by integrating.

Assuming $\hat{f_{i}}(t)$ is the estimated instantaneous frequency of Bragg peak, the frequency shift which caused the ionosphere is

(14) $$\begin{array}{c} {\hat{f}}_{\text{ion}}\left(t\right)=\hat{f_{i}}\left(t\right)-f_{B}, \end{array}$$

where *f*_*B*_ is signed Bragg peak frequency. Phase correction function can be obtained:

(15) $$\begin{array}{c} {\hat{m}}_{\text{ion}}\left(t\right)=-2\pi \overset{t}{\underset{t_{0}}{\int }}{\hat{f}}_{\text{ion}}\left(\tau \right)d\tau . \end{array}$$

Therefore, the results after the phase compensation are as follows:

(16) Rcorr=A(t)ej[θ(t)+m(t)]·e−jm^ion(t)=A(t)ejθ(t)·ej[m(t)−m^ion(t)].

Among them, *A*(*t*) is the echo amplitude, *θ*(*t*) is the phase of echo when the ionosphere is stable, and *m*(*t*) is ionospheric disturbance phase.

### 2.4. Secondary Correction Method to Decontaminate the Ionosphere Based on PGA Algorithm

When phase contamination is serious, two first-order Bragg peaks overlap together. Therefore, we use the S^2^-method to analyze sea clutter contaminated by ionospheric phase perturbation with large amplitude and extract ionospheric frequency modulation from the time-frequency distribution of echo data to sharp the broadening sea clutter spectrum. However, due to the effect of the cross terms, noise and resolution, it is difficult to accurately estimate ionospheric frequency modulation with S^2^-method. However, the broadening spectrum of the Bragg peak is greatly compressed due to the correction; it is easy to separate the two first-order Bragg peak signals. Therefore, we can filter out one of the Bragg peak signals, estimate the residual phase contamination, and improve the final correction performance, which is a cascaded correction method. Here we use phase gradient algorithm (PGA algorithm) [5] to correct residual phase contamination. Because phase contamination becomes lighter after a correction, the PGA algorithm can further sharpen the contamination of the sea spectrum.

## 3. Theoretical Analysis of Suppression Performance

### 3.1. The Performance Analysis of Suppression of Phase Contamination When Spectrum Is Not Overlapped 

After a bandpass filter and IFFT, sea clutter signal of HF skywave radar can be represented as

(17) s(t) =[15exp(j2πfBt)+25exp(−j2πfBt)]·exp(jϕ(t)),

wherein $\sqrt{1/5}$ and $\sqrt{2/5}$, respectively, are the amplitude ratio of the two Bragg peaks of sea clutter and *ϕ*(*t*) is phase contamination function.

**Figure 1:**
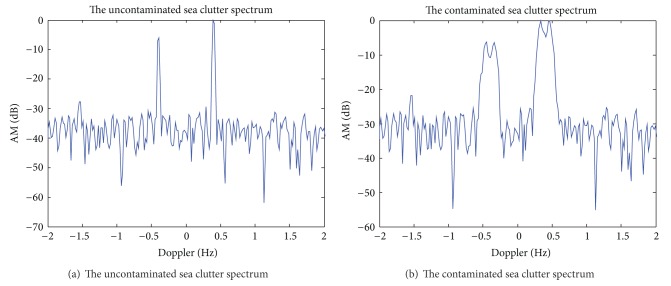
The uncontaminated and the contaminated sea clutter spectra.

**Figure 2:**
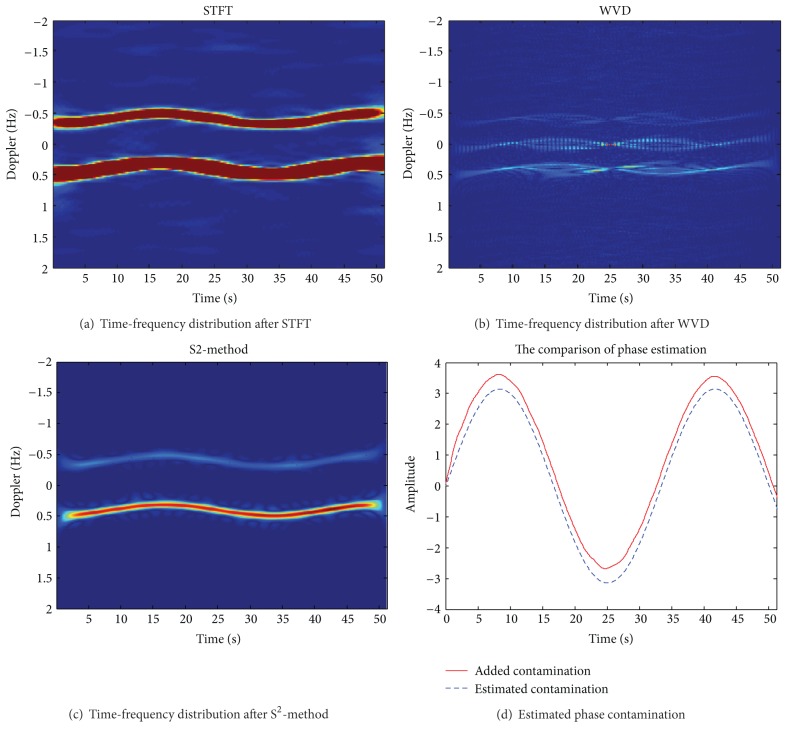
The time-frequency distribution of the contaminated sea clutter after STFT, WVD, and S^2^-method.

Assuming the operating frequency of HF skywave radar *f* = 15 MHz, a coherent accumulation time contains 1024 pulse, pulse repetition period 50 ms, coherent accumulation time *T* = 51.2 s, *L*1 = 16, *L*2 = 2, and SNR = 10 dB.  Figure 1(a) shows the uncontaminated Doppler spectrum of sea clutter. After adding phase disturbance function *ϕ*(*t*) = *π* · sin(2*π* · 0.03*t*), get contaminated Doppler spectrum of sea clutter is shown in Figure 1(b). Figures 2(a)–2(c), respectively, are the time-frequency distribution of the sea spectrum by STFT, WVD, and S^2^-method.  Figure 2(d) shows the extracted phase contamination function from the contaminated sea clutter spectrum by S^2^-method.

The simulation results in Figures 1 and 2 show that the general phase contamination makes the sea clutter become broadening. The time-frequency concentration of STFT is poorer, and cross terms of WVD are very serious, such that it is not conducive to accurately extract the instantaneous frequency. And we can obtain better time-frequency concentration and avoid cross term interference by S^2^-method, and the precision of extraction of phase contamination function can be greatly improved. Sea clutter spectrum also has a good sharpening effect after second correcting ionosphere contamination.

### 3.2. The Performance Analysis of Phase Contamination Suppression When Spectrum Overlaps

**Figure 3:**
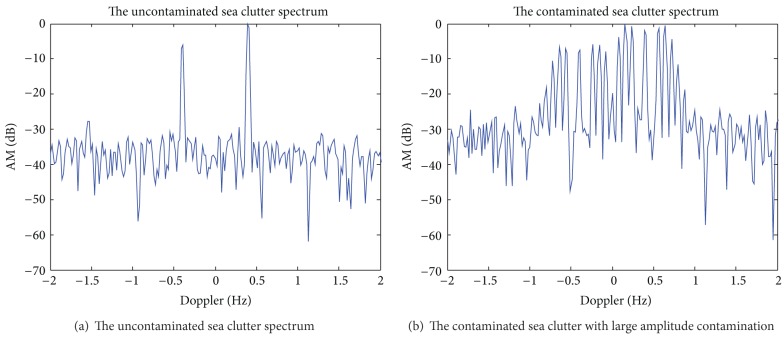
The uncontaminated and the contaminated sea clutter spectra with large amplitude contamination.

**Figure 4:**
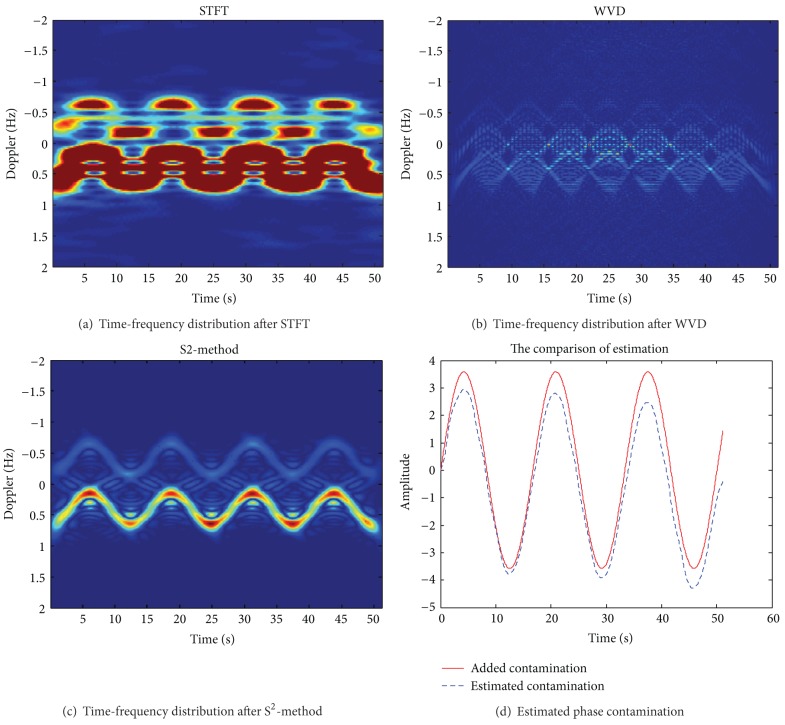
The time-frequency distribution of the contaminated sea clutter with large amplitude contamination after STFT, WVD, and S^2^-method.

The broadening of sea clutter spectrum is not very serious. In Section 3.1, traditional decontamination methods may be used for correction, but these methods all need to bandpass filter for echo spectrum in Doppler domain to extract the positive (or negative) Bragg peak. However, if ionospheric phase perturbation is large, which will make the noise basement of the echo spectrum greatly raised, and two broadening Bragg peaks are difficult to distinguish due to the overlap. So we cannot use band-pass filtering method to extract a Bragg peak. In this case, we cannot use the traditional algorithm of filter Bragg peak to suppress the phase contamination. Figures 3 and 4 show the results of the proposed method in this paper based on S^2^-method cascade processing for the sea spectrum correction with large amplitude contamination.

The simulation parameters are the same as in Section 3.1; contamination function changes into *ϕ*(*t*) = 3.8 · sin(2*π* · 0.08*t*). Figures 3(a) and 3(b) show the original sea spectrum and the contaminated sea spectrum after the severe contamination. Figures 4(a)–4(c), respectively, show the analysis result of STFT, WVD, and S^2^-method for severe contaminated sea spectrum.  Figure 4(d) shows the extracted phase contamination function from the contaminated sea clutter spectrum with large amplitude contamination by S^2^-method.

As can be seen from the simulation results in Figure 3, the large amplitude phase contamination makes two Bragg peaks of sea clutter broaden seriously, and it is hard to distinguish the two broadening Bragg peaks, affecting the target detection (as shown in Figure 3(b)).  Figure 4 shows that the ti me-frequency concentration of traditional STFT distribution is poorer, and cross terms of WVD are very serious, such that it is not conducive to accurately extract the instantaneous frequency. And we can obtain better time-frequency concentration and avoid cross term interference by S^2^-method. After the second correcting ionosphere contamination of sea clutter spectrum also has a good sharpening effect. This method can steadily and effectively sharpen severe sea clutter spectrum contaminated by ionospheric phase perturbation with large amplitude.

### 3.3. The Performance Analysis of Suppression of Phase Contamination under Low Signal-to-Noise Ratio

**Figure 5:**
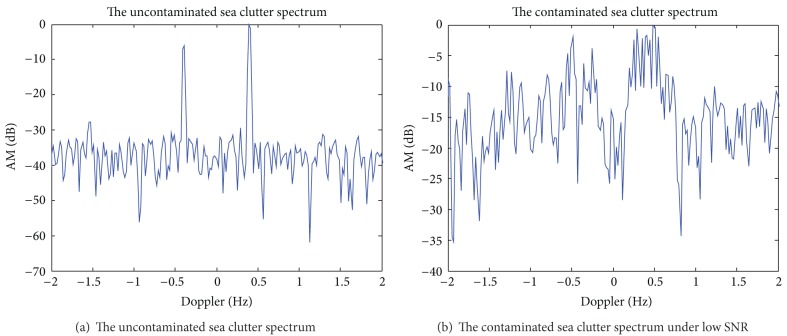
The uncontaminated and the contaminated sea clutter spectra under low SNR.

**Figure 6:**
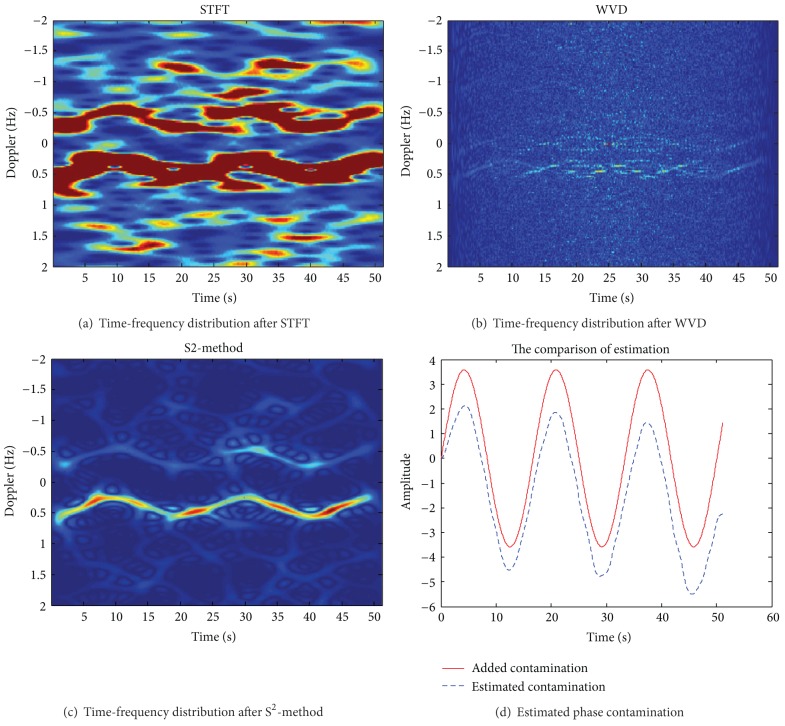
The time-frequency distribution of the contaminated sea clutter with large amplitude contamination under low SNR after STFT, WVD, and S^2^-method.

More time-frequency methods based on the WVD tend to require very strict requirements on signal-to-noise ratio (SNR). There will be greater estimation error when the condition of SNR is not satisfied. Figures 5 and 6 show the results of the proposed method in this paper based on S^2^-method for the sea spectrum correction with large amplitude contamination under low SNR.

Parameter setting: *f* = 10 MHz, the number of frequency modulation cycles is *N* = 1024, SNR = −5 dB, *L*1 = 16, *L*2 = 2, frequency modulation cycle *T* = 50 ms, and ionospheric phase contamination function is *φ*(*t*) = *π* · sin(2*π* · 0.05*t*). Figures 5(a) and 5(b), respectively, show the original sea clutter spectrum and the contaminated sea clutter spectrum under low SNR. Figures 6(a)–6(c) respectively show the analysis result of STFT, WVD, and S^2^-method for contaminated sea spectrum under low SNR.  Figure 6(d) shows the extracted phase contamination function from the contaminated sea clutter spectrum with large amplitude contamination under low SNR by S^2^-method.

From the simulation results in Figure 6, we can see that the S^2^-method method still can relatively accurately extract ionospheric frequency modulation from the time-frequency distribution of contaminated sea clutter at low SNR = −5 dB.

As can be seen from the above results, the approach based on S^2^-method can be used for correcting ionospheric contamination with large amplitude. And compared to the traditional time-frequency analysis methods, this method has high time-frequency resolution, has no cross term interference, and has stronger noise suppression capability than the WVD and STFT.

## 4. The Performance Analysis of Cascaded Approach Based on S^**2**^-Method by Measured Data

**Figure 7:**
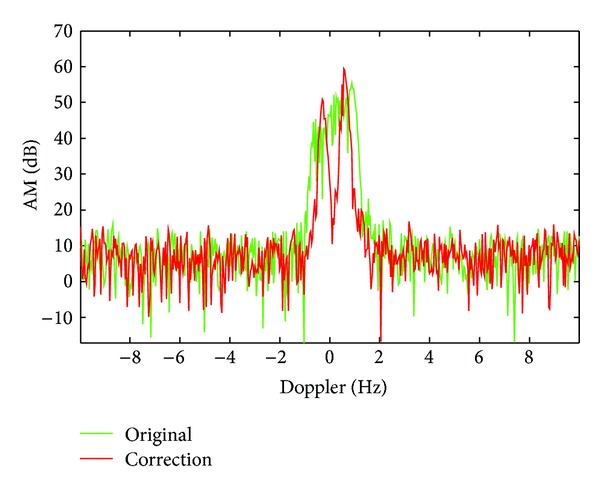
The decontamination results using S^2^-method.

**Figure 8:**
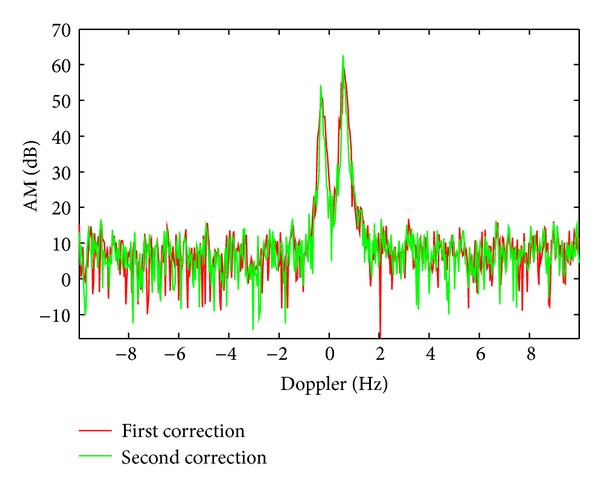
The secondary decontamination results using PGA.

High frequency ground wave radar (HFSWR) and high frequency skywave radar work at the same frequency band, where the structure of sea clutter spectrum is similar. Their difference is that HFSWR has no ionospheric contamination, so artificial adding of phase disturbance can be conveniently compared with the performance of algorithm before and after the correction. Here, we use the measured data of monostatic ground wave over-the-horizon radar. The HFSWR uses the operation mode of linear frequency modulation, the working frequency is *f* = 17.332 MHz, the coherent accumulation time is *T* = 51.2 s, and pulse repetition frequency is PRF = 20 Hz. The added contamination function is *ϕ*(*t*) = *π* · sin(2*π* · 0.05*t*) and the contaminated sea clutter spectrum is shown as the green line in Figure 7. The result of decontamination by S^2^-method is shown as the red line in Figure 7. On this basis, with the method based on PGA, residual phase contamination can be suppressed, which is shown as the green line in Figure 8.

As shown in Figure 7, phase contamination of sea clutter can be suppressed by S^2^-method. On this basis, the effect of decontamination is further improved by PGA method, as shown in the Figure 8 by green line.

Finally, it should be pointed out that we used different data to make many tests for the cascaded correction method and found that this method can always lead to good decontamination effect, which sufficiently shows its robustness. This method is suitable for processing actual complex HF sky wave radar echo with ionospheric contamination.

## 5. Conclusions

This paper proposes a cascaded approach based on S^2^-method to correct the ionospheric phase perturbation with large amplitude. At the first step, time-frequency distribution based on S^2^-method of the time-varying signal is adopted and an optimal detection method is designed to obtain a coarse ionospheric modulation estimation from the time-frequency distribution. At the second step, the residual contamination is eliminated by PGA. Finally, use the measured data to verify the effectiveness of the method. Simulation results show a high time-frequency resolution using this method, which is not affected by the interference of the cross term, ionospheric phase perturbation with large amplitude can be corrected, and the cascade correction method is pretty effective.
